# Stress physiology and weapon integrity of intertidal mantis shrimp under future ocean conditions

**DOI:** 10.1038/srep38637

**Published:** 2016-12-15

**Authors:** Maya S. deVries, Summer J. Webb, Jenny Tu, Esther Cory, Victoria Morgan, Robert L. Sah, Dimitri D. Deheyn, Jennifer R. A. Taylor

**Affiliations:** 1 Scripps Institution of Oceanography, Marine Biology Research Division, University of California at San Diego, La Jolla, CA 92093 USA; 2 Department of Bioengineering, University of California at San Diego, La Jolla, CA 92093, USA

## Abstract

Calcified marine organisms typically experience increased oxidative stress and changes in mineralization in response to ocean acidification and warming conditions. These effects could hinder the potency of animal weapons, such as the mantis shrimp’s raptorial appendage. The mechanical properties of this calcified weapon enable extremely powerful punches to be delivered to prey and aggressors. We examined oxidative stress and exoskeleton structure, mineral content, and mechanical properties of the raptorial appendage and the carapace under long-term ocean acidification and warming conditions. The predatory appendage had significantly higher % Mg under ocean acidification conditions, while oxidative stress levels as well as the % Ca and mechanical properties of the appendage remained unchanged. Thus, mantis shrimp tolerate expanded ranges of pH and temperature without experiencing oxidative stress or functional changes to their weapons. Our findings suggest that these powerful predators will not be hindered under future ocean conditions.

Broad tidal fluctuations in pH and temperature in intertidal habitats are expected to be even greater under the predicted climate change scenarios of ocean acidification and warming^1^. While intertidal organisms may have adaptations that would decrease their susceptibility to changes in ocean chemistry and temperature^2^,^3^, many intertidal calcifiers have demonstrated increased metabolic costs and changes in growth, morphology, and mineralization in response to exposure to increased pCO_2_ and temperature. The intertidal porcelain crab, *Petrolisthes cinctipes*, for example, exhibits increased basal physiological maintenance costs in response to moderate (pCO_2_ = 1,476 μatm, pH 7.6, temperature = 25 °C) and extreme (pCO_2_ = 4,801 μatm, pH 7.1, temperature = 30 °C) changes in pCO_2_ and temperature under simulated tidal fluctuations (hotter at low tide, higher pCO_2_ at high tide)^4^. The periwinkle snail, *Littorina littorea*, shows decreased growth and shell thickness in conjunction with increased metabolic activity, indicating both morphological and stress responses to moderate increases in pCO_2_ (pCO_2_ = 1,000 μatm, pH 7.7)^5^. Some organisms express either a stress or morphological response, but not both. The acorn barnacle, *Semibalanus balanoides,* maintains shell mineral content, but it experiences decreased growth under exposure to moderate and extreme changes in pCO_2_ and temperature (pCO_2_ = 1,000 μatm and 3,000 μatm, pH 7.7 and pH 7.3, temperature = 4.8 °C and 8.5 °C)^6^,^7^. The striped barnacle, *Amphibalanus amphitrite,* however, exhibits increased calcification of its basal plate, yielding higher adhesion strength, but no change in growth when exposed to moderate pCO_2_ levels (pCO_2_ = 2,076 μatm, pH 7.4)^8^. Such responses to ocean acidification and warming can have dramatic ramifications for intertidal ecosystems, as increased energy demands, physiological stress, or changes in mineralization and structural integrity can alter predator defense strategies, competitive ability, and resource gathering^9^. However, changes in structure-function relationships and the effects of such changes on the ecology of organisms and ecosystems have rarely been explored in ocean acidification research^9^.

An extreme example of a highly specialized, calcified structure that might be affected by ocean acidification is the weaponry of mantis shrimp (Crustacea: Stomatopoda). Armed with raptorial appendages that produce one of the fastest, most powerful strikes ever reported in the animal kingdom^10^, mantis shrimp are prodigious predators in intertidal, tropical and subtropical marine ecosystems throughout the world, including coral reefs, mangroves, and sand flats^11^. Some mantis shrimp species, known as “*smashers*,” have hammer-like appendages, which generate high accelerations that yield impact forces of 1,000 N (thousands of times the animal’s own body weight)^12^. These remarkable appendages are not only used to break hard-shelled prey (mollusks, crustaceans) and capture evasive prey (fishes)^13^,^14^,^15^,^16^, but they are also used as weapons during ritualized fighting when animals compete for home cavities^17^. The power of the raptorial appendage is derived from the spring-like properties of the calcified crustacean exoskeleton (Fig. 1). Extensor muscles in the merus segment of the raptorial appendage contract to deform the exoskeleton, storing elastic energy that gets released upon relaxation of the antagonistic flexor muscle, which then rapidly rotates the appendage forward^18^,^19^. The hammer-like striking surface of the appendage called the dactyl club is heavily mineralized and able to withstand high impact forces (Fig. 1)^20^,^21^. Thus, the structure, composition, and mechanical properties of the exoskeleton are critical for proper functioning of this potent weapon.

In addition to specialized functions, the exoskeleton plays other critical roles in almost every aspect of crustacean biology and ecology (locomotion, defense, communication, and support for reproduction and growth). This impressive functionality is derived from the architecture of the cuticular exoskeleton, which is a composite material made primarily of a protein-chitin matrix embedded with mineral^22^. The cuticle is subdivided into four distinct layers, from the exterior to interior: epicuticle, exocuticle, endocuticle, and membranous layer^22^. All layers, except the membranous layer, are calcified, typically with calcium carbonate in the form of crystalline calcite or amorphous calcium carbonate, but magnesium and phosphorous are also present in smaller amounts^23^,^24^. While the matrix provides the cuticle with fracture toughness (resistance to crack propagation), the mineral provides hardness (resistance to permanent deformation) and stiffness (rigidity or resistance to deformation)^22^. Crustaceans manipulate multiple aspects of the cuticle, such as layer thickness, mineral composition, and the mineral to matrix ratio, to fine-tune mechanical properties such as stiffness, hardness, and strength^21^,^22^,^25^. Changes to mineralization patterns in response to ocean acidification can therefore affect the mechanical properties and functioning of the crustacean exoskeleton in profound ways.

Thus far, there have been comparatively few studies on the effects of ocean acidification and warming conditions on crustacean mineralization, but there appears to be mixed responses that are possibly related to differences in thermal ranges and habitat requirements^26^. Some crustaceans demonstrate altered exoskeleton mineralization (increased calcification in the striped barnacle *Amphibalanus amphitrite*^8^ and caridean shrimp *Lysmata californica*^27^; decreased calcification in the tanner crab *Chionoecetes bairdi*^28^; increased magnesium in the velvet swimming crab *Necora puber*^29^), while others show no change (acorn barnacle *Semibalanus balanoides*^6^,^7^, American lobster *Homarus americanus*^30^, blue crab *Callinectes sapidus*^30^, and eastern king prawn *Penaeus plebejus*^30^). However, the lack of measureable changes to the exoskeleton are often accompanied by an increase in metabolic activity or a decrease in growth^6^,^7^,^28^.

The variability in responses to ocean acidification is generally attributed to the physiology of calcification in crustaceans, as bicarbonate ions (HCO_3_^−^) and metabolic CO_2_ may be used in the process of calcification as opposed to carbonate ions^25^,^26^,^30^,^31^,^32^, thereby making crustaceans more tolerant to low pH conditions. Additionally, crustaceans have an acid-base regulatory system that effectively buffers alterations to hemolymph pH, which might protect exoskeleton construction from external changes in ocean carbon chemistry^3^,^29^,^33^,^34^,^35^. Despite having this regulatory ability, crustaceans appear to experience increases in enzymatic activity and lipid oxidation (indicators of oxidative stress) when the acid-base regulatory system is active, which could lead to metabolic depression over the long-term^36^,^37^,^38^.

Raptorial appendage function in mantis shrimp depends on animal physiology and on the structure and composition of the exoskeleton. How changes in mineralization specifically affect the morphology and mechanical properties of the crustacean exoskeleton remains poorly studied despite their critical importance to animal function. This is true for other marine calcifiers as well. In one of few examples, the oyster, *Crassostrea virginica,* demonstrates an increase in shell thickness, but a reduction in shell fracture toughness and hardness under extreme increases in pCO_2_ (pCO_2_ = 3,500 μatm, pH 7.5), thereby increasing the susceptibility of this species to predation and parasites^39^. If forecasted changes in ocean carbon chemistry can similarly alter the morphology and mechanical properties of the mantis shrimp exoskeleton, then it could potentially affect the raptorial strike and other behaviors, ultimately causing cascading effects throughout the ecosystems that mantis shrimp inhabit. Yet, to date, no mantis shrimp, and relatively few crustaceans, have been focal in ocean acidification research.

Our goal was to examine the long-term effects of moderate increases in pCO_2_ and temperature on mantis shrimp stress physiology, as well as the exoskeleton morphology and mechanical properties of the merus segment of the raptorial appendage as compared to the carapace, which is a less specialized segment of exoskeleton. Specifically, for three and six months, we examined the intertidal coral reef predator, *Neogonodactylus bredini* (Manning, 1969), under near-future scenarios of pCO_2_ and temperature for coastal regions following models of the Intergovernmental Panel on Climate Change (IPCC) and a coastal oceanographic study^40^. The feeding mechanics and ecology of this species are relatively well-understood^13^,^19^. We predicted that reduced pH combined with increased temperature would elicit an oxidative stress response in *N. bredini* as well as an increase in exoskeleton mineralization, yielding a stiffer, harder exoskeleton that would make the raptorial appendage more brittle and less effective. To our knowledge, this is the first study to examine the effects of ocean acidification and warming on multiple levels of organismal organization, from physiological process at the cellular level to exoskeleton composition, morphology, and mechanical properties.

## Results

### Molting and growth

All animals survived the experiment except for one individual from the reduced pH treatment. From the three-month exposure, 13 individuals molted once (six, five, and four individuals in the ambient, reduced pH, and reduced pH/increased temperature treatments, respectively). From the six-month exposure, all but two of the largest individuals molted; seven individuals molted twice, five of which were from the reduced pH/increased temperature treatment and two of which were from the ambient and reduced pH treatment. Animals that molted twice ranged in carapace length from 7.95–11.60 mm, which spanned most of the animal size range (7.95–12.27 mm) and demonstrates that both large and small animals underwent multiple molts.

Measurements of growth (percent change in mass and percent change in carapace length) were not significantly different between treatments. The mean ± standard deviation percent mass growth for the reduced pH and the reduced pH/increased temperature treatments were 2.43 ± 0.7% (*N* = 20) and 2.59 ± 0.95% (*N* = 18), respectively, which were not significantly different from that of the ambient treatment (2.58 ± 1.02%, *N* = 19) (one-way ANOVA: *P* = 0.81, *F*_2.54_ = 0.22, *N* = 57) across both the three-month and six-month exposure periods. Percent growth in carapace length was also not significantly different between the treatments (ambient: 7.31 ± 6.91%, *N* = 21; reduced pH: 6.56 ± 5.27%, *N* = 22; reduced pH/increased temperature: 5.51 ± 5.11%, *N* = 24; one-way ANOVA: *P* = 0.63, *F*_2,64_ = 0.47, *N* = 67). Both measurements of growth varied with animal size, with smaller animals growing more than larger animals (least-squares linear regression: percent change in mass: slope = −2.86, *P* = 9.18 × 10^−6^, *F*_1,55_ = 23.9, *N* = 57, percent change in carapace length: slope = −4.54, *P* = 3.64 × 10^−8^, *F*_1,65_ = 39.01, *N* = 67) (Fig. 2). However, the slopes for each treatment were not significantly different from one another (ANCOVA: percent change in mass: *P* = 0.81, *F*_2,54_ = 0.22, *N* = 57; percent change in carapace length: *P* = 0.63, *F*_2,64_ = 0.47, *N* = 67) (Fig. 2).

### Oxidative stress

We examined protein damage due to oxidative stress as well as the activity levels of two enzymes that are produced in response to oxidative stress, superoxide dismutase (SOD) and catalase (CAT), as proxies for induced oxidative stress in individuals. The range of protein concentrations across treatments was 0.5–2.5 μg/μL. There were no significant differences in the concentrations of proteins with carbonyl bonds between treatments (Kruskal-Wallis: *P* = 0.22, *χ*^*2*^_4_ = 5.73, *N* = 16), nor were there differences in SOD concentrations (one-way ANOVA: *P* = 0.20, *F*_4,11_ = 1.79, *N* = 16) or in CAT concentrations (Kruskal-Wallis: *P* = 0.31, *χ*^*2*^_4_ = 4.79, *N* = 16) (Table 1).

### Cuticle structure

Scanning electron microscopy (SEM) imaging revealed no visual differences in the cuticle structure between the three treatments for either the merus or carapace cuticle samples (Fig. 3). In both the merus and carapace, the epicuticle and procuticle (exocuticle and endocuticle) layers were distinguishable and consistent in relative thickness, with lamellae stacking density greatest in the exocuticle (Fig. 3). The membranous layer was not visible in either the merus or the carapace.

Body size-corrected total thickness (epicuticle, exocuticle, and endocuticle) of the cuticle, as measured from the SEM images, was not significantly different between treatments or between the exposure periods (three-way ANOVA: treatment: *P* = 0.33, *F*_2,99_ = 1.11; exposure period: *P* = 0.82, *F*_1,99_ = 0.056, *N* = 111), but the carapace cuticle was significantly thicker than the merus (mean difference [95% confidence interval]: 1.40 [0.7, 1.99], *P* = 6.16 × 10^−5^, *F*_1,99_ = 17.52, *N* = 111) (Table 2, Fig. 4).

### Mineralization patterns

The weight percent of key elements involved in exoskeleton construction, Ca and Mg, were measured using energy-dispersive X-ray spectroscopy (EDX). There were no significant differences in % Ca between treatments (three-way ANOVA: *P* = 0.78, *F*_2,100_ = 0.25, *N* = 105; Fig. 5). Within both the merus and carapace, exoskeleton from the six-month exposure had greater % Ca compared to that from the three-month exposure (merus: mean difference = 8.61 [4.66, 12.56]%, *P* < 0.0001, *F*_1,49_ = 19.19, *N* = 53; carapace: mean difference = 5.06 [1.08, 9.04]%, *P* = 0.01 *F*_1,48_ = 6.55, *N* = 52) (Table 2, Fig. 5).

There were significant differences in % Mg between treatments (three-way ANOVA: *P* = 0.006, *F*_2,100_ = 5.39, *N* = 105) (Table 2, Fig. 5). A Tukey’s posthoc test revealed that the % Mg in the reduced pH treatment was significantly higher than that of the ambient and the reduced pH/increased temperature treatments (reduced pH - ambient: mean difference = 0.32 [0.02, 1.00]%, Tukey adjusted *P* = 0.04; reduced pH - reduced pH/increased temperature = 0.56 [0.08, 1.04]%, Tukey adjusted *P* = 0.02). When differences between treatments were examined within body segments, this pattern was only present in the merus and not in the carapace (two-way ANOVA: merus: *P* = 0.01, *F*_2,49_ = 4.86, *N* = 54; carapace: *P* = 0.28, *F*_2,48_ = 1.32, *N* = 53) (Table 2, Fig. 5).

There were no significant differences in Ca:Mg between treatments (three-way ANOVA: treatment: *P* = 0.08, *F*_2,93_ = 2.55, *N* = 104; Fig. 5). Within the merus, Ca:Mg was higher in the six-month exposure compared to the three-month exposure (two-way ANOVA: mean difference = 4.23 [0.72, 7.74], *P* = 0.01, *F*_1,49_ = 7.09, *N* = 52), but not within the carapace (*P* = 0.50 *F*_1,48_ = 1.50, *N* = 51).

EDX elemental mapping of one individual from each treatment from the six-month exposure showed that both Ca and Mg were evenly distributed across the merus and the carapace cuticle layers, with no clear differences between treatments (Supplementary Fig. S1).

Volume renders of the Micro-CT scanned samples (Fig. 6) support the EDX data, showing no differences in cuticle mineralization between treatments for the carapace, merus, or dactyl club (Table 3). Mineralization of both the merus and carapace was relatively uniform. The dactyl club exhibited a highly mineralized region in the outer layer, but the inner layer was less mineralized (Table 3). Although we only examined one individual per treatment, the mineral density regions that were quantified for each body segment did not appear to be different between these representative individuals (Table 3).

### Mechanical properties

The hardness and stiffness of the merus and carapace cuticle were measured using a nanoindentation materials testing machine. The maximum penetration depth of the indenter tip into the exoskeleton was 3.57 ± 0.27 μm, but the mean thickness of the epicuticle was 4.49 ± 1.9 μm, which indicates that we primarily tested the outer region of the cuticle.

There was no treatment effect on carapace hardness (two-way ANOVA: *P* = 0.36, *F*_2,49_ = 1.05, *N* = 52) or stiffness (two-way ANOVA: *P* = 0.70, *F*_2,49_ = 0.37, *N* = 52). However, both variables were greater at six months compared to at three months (hardness: two-way ANOVA: mean difference: 0.11 [0.05, 0.17] GPa; *P* = 0.0006, *F*_1,49_ = 13.42, *N* = 52; stiffness: mean difference: 3.50 [1.50, 5.50] GPa; *P* = 0.0009, *F*_1,49_ = 12.42, *N* = 52) (Table 2, Fig. 7).

Within the merus, we found no significant differences in hardness between treatments or exposure periods (two-way ANOVA: treatment: *P* = 0.76, *F*_2,50_ = 0.26, *N* = 56; exposure period: *P* = 0.07, *F*_1,50_ = 3.46, *N* = 56), nor did we find differences in stiffness (two-way ANOVA: treatment *P* = 0.64, *F*_2,50_ = 0.46, *N* = 56; exposure period *P* = 0.72, *F*_1,5o_ = 0.13, *N* = 56) (Table 2, Fig. 7).

### Influence of molting

The time since the last molt was used to determine if the molting process affected any of the response variables (i.e. growth, thickness, % Ca, % Mg, Ca:Mg, hardness, and stiffness), and no significant effect was detected (all Bonferroni adjusted *P*-values > 0.004). We were specifically interested in this comparison for % Mg, given that we found a significant difference in % Mg between treatments in the merus. However, there was no significant effect of the time since the last molt on % Mg (ANCOVA: *P* = 0.03, *F*_1,48_ = 4.88, *N* = 54).

## Discussion

To our knowledge, this study is the first integrative analysis of the effects of ocean acidification and warming on mantis shrimp and calcified weaponry. Most intertidal organisms exhibit a negative response to reduced pH in one, if not all, of the physiological and morphological traits that are commonly measured. In contrast, *N. bredini* showed no changes in growth, molting, enzymatic and protein indicators of oxidative stress, exoskeleton morphology, calcium content, or mechanical properties in response to experimental pH and temperature stressors, suggesting that this species has evolved compensation mechanisms to cope with significant environmental change. This study also revealed that, unlike in the carapace, increases in % Ca over time in the merus did not lead to corresponding changes in mechanical properties. Here, we discuss *N. bredini’s* apparently large tolerance range for changes in environmental pH and temperature as it relates to stress physiology and raptorial appendage structure and function. We then consider these findings in the context of *N. bredini* remaining an effective intertidal coral reef predator and competitor in the face of global climate change.

### Mantis shrimp showed no evidence of oxidative stress under ocean acidification conditions

Most animals that are considered tolerant to ocean acidification and warming conditions predicted for the next century demonstrate acid-base regulation strategies^3^,^34^, which are often accompanied by increased metabolic or enzymatic activity^36^. While we did not explicitly examine acid-base regulation, we argue that no increase in SOD and CAT activity, coupled with no differential protein damage in the form of carbonyl-bond addition, indicate that animals were functioning within their physiological tolerance range in both the reduced pH and reduced pH/increased temperature treatments. Expression of genes coding for SOD and CAT has been shown to increase in oysters and stenoecious crabs in response to exposure to ocean acidification and warming^36^,^41^. However, unpublished transcriptome data from *N. bredini* in this experiment support our findings, as the gene expression of SOD and CAT was also indistinguishable between treatments (N. T. Pierce and M. S. deVries, unpublished data).

Typically, acute and drastic decreases in pH elicit reduced growth, increased metabolic activity, cellular damage, or even mortality in crustaceans^26^,^38^. Yet, even in a short-term exposure to seawater with a low pH of 7.3, levels of SOD, CAT, and protein damage were statistically similar to those from individuals kept in ambient pH (7.9) (Table 1). In fact, the concentration of proteins with carbonyl bonds was numerically higher in the ambient treatments compared to the other treatments (Table 1), suggesting that *N. bredini* might experience less stress under reduced pH conditions. Thus, if the animals were using acid-base regulation to compensate for changes in internal pH, this mechanism did not yield sufficient metabolic activity to generate high levels of ROS that would induce an enzymatic stress response or damage proteins. Exposure to reduced pH and reduced pH/increased temperature yielding no effects on SOD and CAT levels or protein damage further suggests that mantis shrimp have wide tolerances to high pCO_2_ and high temperature environments.

*N. bredini*’s impressive tolerance may have evolved because the pH ranges experienced in this species’ intertidal habitat are quite wide (pH 7.9–8.4, Supplementary Table S1). However, this does not explain why *N. bredini* demonstrated tolerance to a pH of 7.3, which is far below the range experienced in its natural habitat. Given that we provided the animals with ample food, albeit of only one prey type (i.e., mole crabs), it is possible that the mantis shrimp were not energetically limited, thereby mediating their response to the extremely low pH level. Abundant food supply confers tolerance to low pH conditions for a variety of other invertebrates, including corals, mussels, barnacles, crabs, copepods, and sea urchins (reviewed in ref. 42). Potential changes in the food supply of *N. bredini* may impact their diet and affect their tolerance to ocean acidification in turn.

Selection pressure for the evolution of a wide tolerance range could have also stemmed from *N. bredini*’s cavity dwelling lifestyle. Specifically, respiration may have a large impact on increasing CO_2_ and correspondingly decreasing pH in *N. bredini*’s coral rubble cavity. During the heat of the day, at night, and throughout molting, individuals remain in their coral rubble cavities, often blocking the entrance with a rock^13^, which restricts water flow in the cavity. In the laboratory, *N. bredini* individuals housed in 0.2 L cups of water with no flow can decrease ambient pH by 0.2 within 24 hours^43^. With an even smaller volume of water in a coral rubble cavity, *N. bredini* could drastically alter the local carbonate chemistry and therefore experience and adapt to wider fluctuations than that of their surrounding environment.

### Cuticle % Ca did not change in response to ocean acidification conditions

Concomitant with no apparent stress response, *N. bredini* did not exhibit changes in exoskeleton calcification patterns under experimental pH and temperature stressors. Neither the carapace nor the merus segment of the raptorial appendage demonstrated changes in % Ca under reduced pH and reduced pH/increased temperature treatments. This response is unlike that of many other calcified organisms that display either decreases or increases in calcification^3^,^26^,^28^,^44^,^45^. Similar to mantis shrimp, however, other crustaceans (blue crabs, paneid shrimp, and American lobsters) and echinoderms^30^,^31^,^45^ show no net change in calcification. For acorn barnacles and tanner crabs, this response comes at the expense of growth, metabolic investment, and/or physiological stress^6^,^7^,^28^. Thus, experiencing no net change in calcification under reduced pH conditions may be a relatively common response in crustaceans, but too few studies have been conducted to determine if this response is typically accompanied by stress.

When exposed to ocean acidification conditions, crustaceans must contend with undersaturation of calcite in seawater, which promotes dissolution^26^. The epicuticle in crustaceans may serve as a protective layer against changes in external seawater conditions, thereby preventing dissolution^31^. At the same time, crustaceans use acid-base regulation to buffer hemolymph pH from increases in seawater HCO_3_^−^ so that hemolymph pH, and therefore the availability of HCO_3_- for exoskeleton construction in hemolymph, is maintained^3^,^29^,^31^,^33^,^34^,^35^. These mechanisms could be working in concert to help mantis shrimp maintain the calcification patterns of their exoskeleton under reduced pH conditions.

### Unlike the carapace, the raptorial appendage maintains its integrity over time

While there was no change in % Ca in response to reduced pH, it was significantly higher at six months compared to three months in both the merus (8.6% greater) and carapace (5.1% greater), demonstrating a time component for accruing exoskeleton Ca. This increase over time could have been due to the timing of the molt cycle, which can last three to four months in *N. bredini*^46^, but we sampled individuals at least 18 days following ecdysis (i.e., shedding of the exoskeleton), which is sufficient time for the newly formed exoskeleton to fully harden^46^. Indeed, there was no correlation between % Ca and the time since ecdysis (range: 18–171 days). Thus, the observed increase in % Ca reflects long-term accretion rather than deposition related to the molt cycle. With the observed increases in % Ca over time, we had expected corresponding changes in mechanical properties, which are dependent on both the mineral composition and structure of the exoskeleton^22^,^25^. We found that the carapace was on average 0.11 GPa harder and 3.50 GPa stiffer across treatments at six months compared to three months. Thus, even relatively small changes in % Ca can coincide with significant changes in exoskeleton mechanical properties of the carapace.

**Figure 1:**
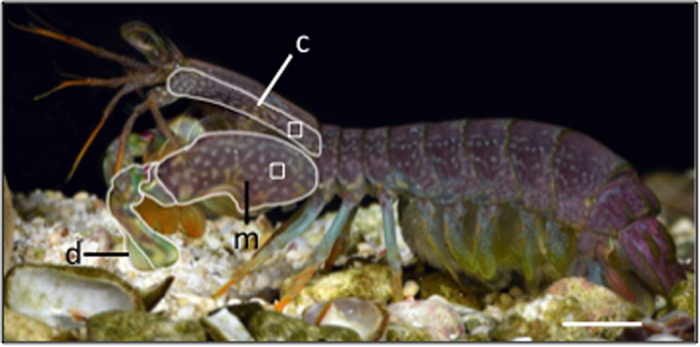
Adult *Neogonodactylus bredini* showing regions of the body analyzed in this study. The individual is in lateral view with the merus (m) and dactyl (d) of the left raptorial appendage and left carapace flap (c) outlined. Scanning electron micrographs of the cuticle in cross-section were taken along the merus and carapace where mineralization was also quantified. The boxes on the merus and carapace represent locations where nanoindentation occurred for materials testing. Micro-CT scans of the carapace, merus, and dactyl were also taken. Scale bar = 5 mm. Image courtesy of R. L. Caldwell and modified from^16^.

**Figure 2:**
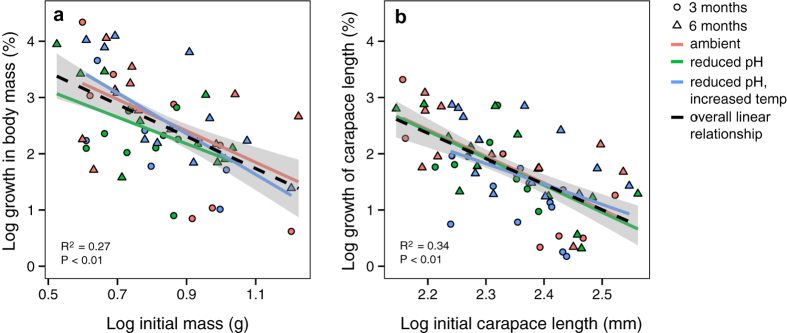
Growth. Growth parameters (**a**) percent change in mass [*N* = 57] and (**b**) percent change in carapace length [*N* = 67]) of the individuals from the three-month (circles) and six-month (triangles) exposures show that percent growth changed as expected over time; the smaller animals showed a greater increase in percent growth compared to the larger animals. R^2^ values describe the overall least-squares linear regression models with no treatment affect (black lines) and the grey shading represents the 95% confidence intervals of the models.

**Figure 3:**
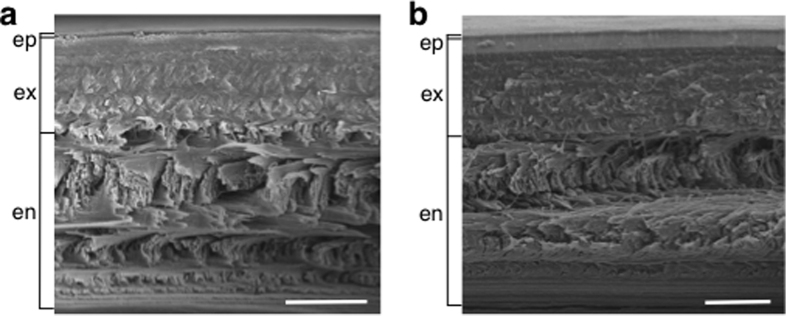
Representative scanning electron micrographs of the cuticle. The (**a**) merus cuticle and (**b**) carapace cuticle are shown. Cuticle layers and their approximate thicknesses are noted on the left. There were no visible differences in cuticle morphology between the treatments (*N* = 111). The membranous layer of the cuticle was not visible. ep = epicuticle, ex = exocuticle, en = endocuticle. Scale bars = 20 μm.

**Figure 4:**
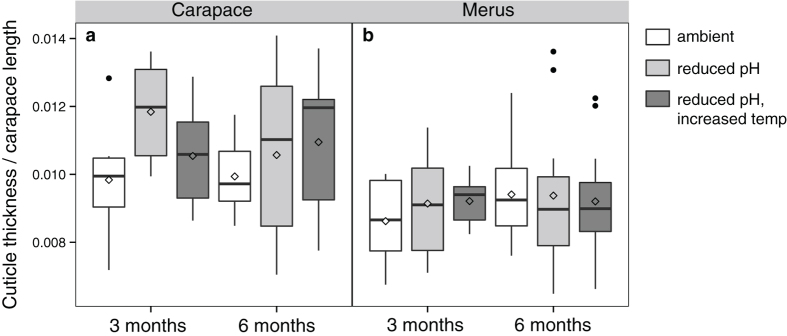
Total cuticle thickness. Box plots (box boundaries = the 25^th^ and 75^th^ quartiles, error bars = 1.5 times the interquartile distance, center line = median, diamond = mean) illustrate that no significant differences in mean total cuticle thickness in mm divided by carapace length in mm (to correct for body size) were detectable between treatments either within the (**a**) carapace or (**b**) merus or between the three- and six-month exposure periods (*N* = 111). Black dots represent outliers from the box plots, but values were included in the analysis because there were no obvious biological reasons to exclude them.

**Figure 5:**
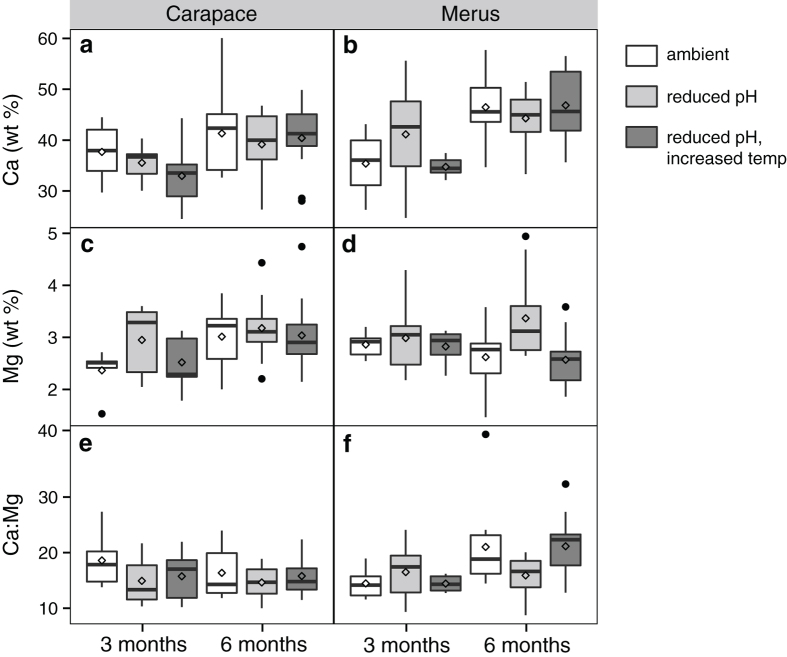
Cuticle mineralization. Box plots of the mineralization content (presented as weight percent calcium [Ca] and weight percent magnesium [Mg]; box boundaries = the 25^th^ and 75^th^ quartiles, error bars = 1.5 times the interquartile distance, center line = median, diamond = mean) in the cuticle for (**a**,**c**,**e**) the carapace and (**b**,**d**,**f**) merus from the three- and six-month exposure periods (*N* = 105). (**a**,**b**) Ca, (**c**,**d**) Mg, and (**e**,**f**) the ratio of Ca:Mg. Mg in the merus from the reduced pH treatment, in particular, is significantly different from the other treatments. Black dots represent outliers from the box plots that were included in the analyses.

**Figure 6:**
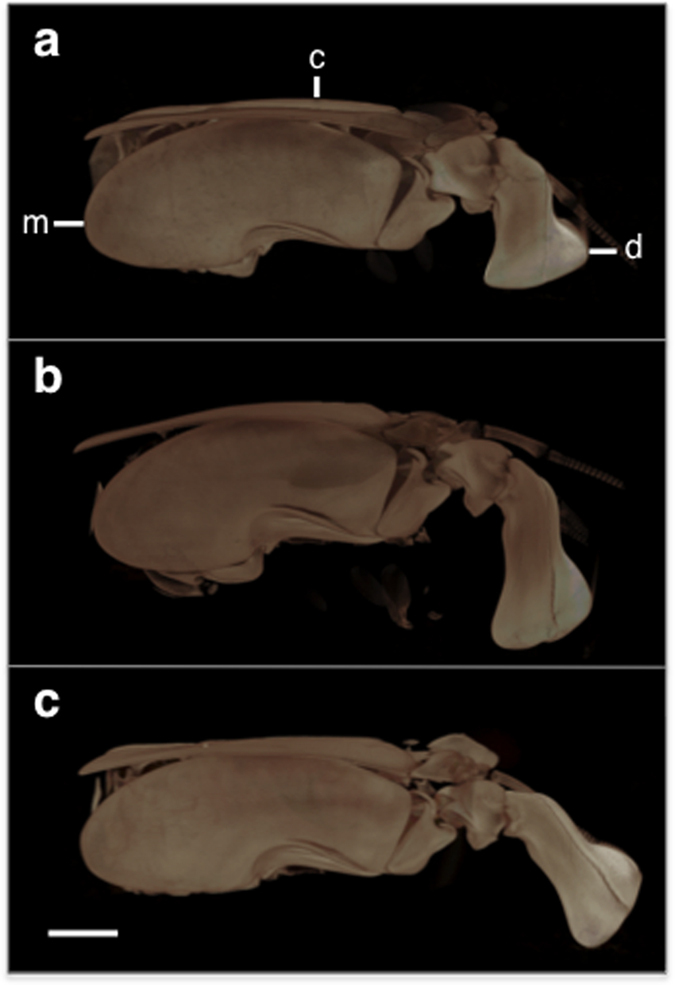
Micro-Computed Tomography (Micro-CT) 3D volume renders of the raptorial appendages. The lateral sides of the appendages are shown from the (**a**) ambient, (**b**) reduced pH, and (**c**) reduced pH/increased temperature treatments and show no obvious differences in calcification patterns between treatments (*N* = 3). The anatomical features from which the mineralization data were recorded are labeled as followed: merus (m), carapace (c), dactyl (d) (see Supplementary Fig. S4 for detailed depictions of the analyzed locations). Scale bar = 2.5 mm.

**Figure 7:**
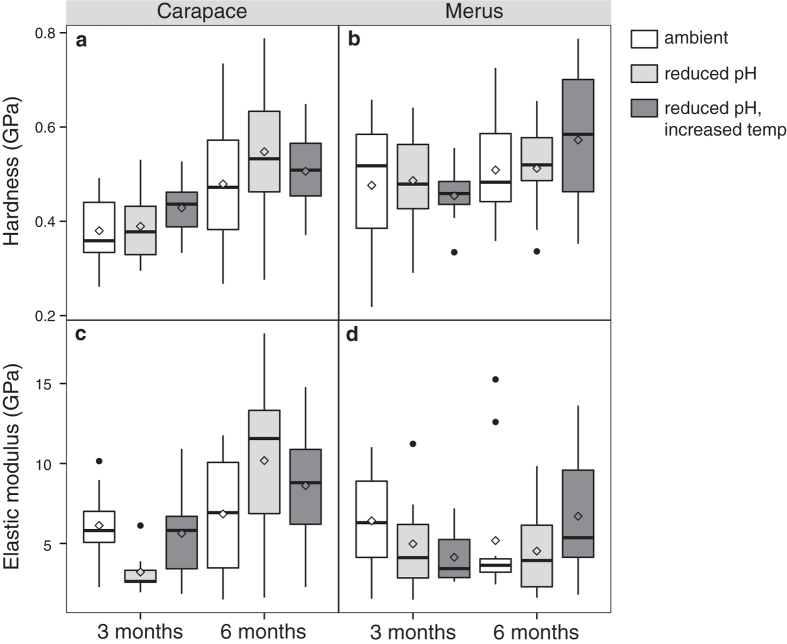
Cuticle mechanical properties. Box plots (box boundaries = the 25^th^ and 75^th^ quartiles, error bars = 1.5 times the interquartile distance, center line = median, diamond = mean) of (**a**,**b**) hardness and (**c**,**d**) elastic modulus (i.e. stiffness) of the (**a**,**c**) carapace and (**b**,**d**) merus show that the mechanical properties of the merus are maintained over time across treatments, but the carapace increased in hardness and elastic modulus (stiffness) across treatments between the three- and six-month exposure periods (*N* = 111). Black dots represent outliers from the box plots that were included in the analyses.

The merus exoskeleton, however, produced surprising results. Even though the merus exhibited a larger increase in % Ca at six months compared to the carapace, both hardness and stiffness remained constant. This result may be related to the specialized functions of the merus. While the carapace is essential for basic defense and structural support, the merus has the added function of producing fast, powerful movements. Maintaining the mechanical properties of the merus exoskeleton is critical because its kinematics depend on the exoskeleton behaving like a spring^10^,^18^,^19^. These spring-like properties are determined partly by the stiffness of the appendage exoskeleton^18^,^19^,^47^, to which Mg and Ca contribute^21^,^24^,^47^. Due to sampling limitations, we did not measure the specialized spring components of the merus, but rather a region proximal to these components (Fig. 1). Yet, this region of the merus still revealed an unexpected relationship between mineral content and mechanical properties. Crustaceans can construct cuticle for different functions through a variety of mechanisms, such as morphological differences in cuticle layer formation and structural differences in calcite crystal formation^20^,^21^,^22^,^25^,^48^. Further research comparing the ultrastructure of the merus and carapace would help to uncover the mechanisms underpinning how *N. bredini* is able to maintain the mechanical properties of the merus despite changes in % Ca.

Merus cuticle is further distinguishable from that of the carapace by the addition of Mg under reduced pH conditions; % Mg was 0.32–0.56% higher in the reduced pH treatment across exposure periods. A similar result was documented in the velvet crab, *Necora puber*, which showed increased amounts of Mg in the chelae and hemolymph, but not in the carapace, in response to extreme pH reductions (pCO_2_ = 1,100 μatm and 9,000 μatm, pH 7.7 and pH 7.3)^29^. Taken together, these results show that some crustaceans incorporate elements differently between regions of the exoskeleton in response to reduced pH, but the mechanisms behind these patterns are not clearly understood and require further study.

While the merus segment of the raptorial appendage functions like a spring, the dactyl club must withstand the high impact forces experienced during contact with hard prey items and ritualized fighting with conspecifics^17^. These behaviors require the dactyl club to be both hard and tough. The distribution and amount of calcium and other minerals in the striking surface of the dactyl segment of the appendage are closely tied to the dactyl’s ability to withstand high impacts from striking^21^,^24^. Specifically, the impact surface consists of hard hydroxyapatite crystallites and amorphous calcium phosphate and carbonate, which contribute to the hardness and stiffness of this part of the appendage^21^. While we did not examine the elemental composition or mechanical properties of the dactyl, mineral densities quantified from Micro-CT scans suggest that there were no differences in dactyl mineralization under reduced pH conditions. There were also no obvious differences between treatments in Micro-CT quantified mineral density in either the merus or the carapace (in the six-month exposure), thereby providing further support for our conclusions derived from EDX. Additionally, it appears that the slight change in % Mg in the merus does not alter measures of mineral density as detected by Micro-CT.

The differences in mineral content and mechanical responses of distinct regions of the exoskeleton, along with variation in Mg due to reduced pH, demonstrate the need to address the complexity of the exoskeleton in ocean acidification research. Specifically, more detailed study of the construction and ultrastructure of multiple regions of the exoskeleton under experimental pH conditions would help to elucidate how crustaceans respond to ocean acidification and warming, and would be especially informative when coupled with study of the associated cellular physiology.

### The combined stressors of ocean acidification and warming do not act synergistically

Ocean acidification is occurring simultaneously with ocean warming, and together these environmental stressors can intensify the effects of ocean acidification on organisms^49^. For example, reduced pH negatively impacts immune function, lipid oxidation, and cellular damage in the Norway lobster, *Nephrops norvegicus*, but the combined effects of pH and temperature significantly increases the severity of these impacts (pCO_2_ = 857–1,725 μatm, pH 7.7–7.5, temperature = 5–18 °C)^38^. Negative effects of reduced pH on the growth rate of the sea star, *Asterias rubens*, are enhanced by increases in temperature^50^. Few studies to date have examined the combined effects of ocean acidification and warming on the mineralization and mechanical properties of calcified structures, but there does seem to be a synergistic effect. Structural disorientation of calcite crystals in the shells of the mussel, *Mytilus edulis,* increased dramatically under combined pH/temperature treatments compared to reduced pH alone, which possibly decreased the structural integrity of the shell (pCO_2_ = 750 μatm and 1,000 μatm, pH 7.5 and pH 7.2, temperature = 10 °C and 12 °C)^48^. In mantis shrimp, the main treatment response (i.e. higher % Mg in the merus) occurred in the reduced pH treatment but not in the combined reduced pH/increased temperature treatment. These results suggest that the combination of pH and temperature mitigated the, albeit small, effects of reduced pH alone.

### With no changes to physiological stress or mechanical integrity of the exoskeleton, mantis shrimp appear armed to face global climate change

Upholding the integrity and functionality of the raptorial appendage without a heightened oxidative stress response is crucial for the survival of *N. bredini* because raptorial appendages are used for three critical functions: feeding, fighting and defense^17^. Having a high tolerance for environmental fluctuations, in terms of both stress and morphology, is advantageous for *N. bredini* in a dynamic intertidal environment where a diurnal change in pH of 0.4 (Supplementary Table S1) occurs regularly and temperature can also rise dramatically. Although we did not fluctuate pH and temperature conditions in our experiment (the impact of which is uncertain), it is clear that mantis shrimp can tolerate constant low pH values at or below the forecasted predictions for the next century. Mantis shrimp must be able to cope with daily fluctuations in pH and temperature, while simultaneously settling disputes with conspecifics^51^ and crushing hard-shelled prey that are energetically costly to consume^13^,^52^. Thus, having the ability to cope with wide ranges of pH and temperature while maintaining raptorial appendage structure and function allows *N. bredini* to devote resources to feeding and to predator and territory defense.

The exact mechanisms through which *N. bredini* copes with stress and maintains the integrity of the raptorial appendage, from the specialized spring to the hard smashing surface of the dactyl club, in the face of ocean acidification and warming conditions have yet to be determined. Investigating these mechanisms, however, presents an exciting opportunity to understand the tolerance of intertidal organisms to future climate change. For example, targeting physiological characteristics that make *N. bredini* robust to predicted ocean conditions, such as stress regulation and internal acid/base regulation, would yield new insights into how intertidal organisms may adapt to future changes in ocean conditions. We therefore view *N. bredini* as a model system for understanding why and how some organisms can better tolerate simulated ocean acidification and warming while others cannot. It is also widely recognized that the early developmental stages of crustaceans, such as tanner crabs, lobsters, shrimp, and krill, can be detrimentally effected by high pCO_2_ conditions and have carry-over effects on later life history stages^53^,^54^,^55^,^56^,^57^. Adult mantis shrimp could be similarly affected by their past environmental history, which merits further study. Moreover, mantis shrimp live in diverse habitats throughout tropical and subtropical regions in depths ranging from 0–900 m^11^. Studying the generalities of our findings across mantis shrimp taxa may reveal novel compensation mechanisms that would help to explain the evolution of tolerance in mantis shrimp and possibly other organisms as well.

Overall, this study highlights the importance of extending ocean acidification research beyond the level of morphology to incorporate the ecologically critical aspects of mechanical properties and functionality of animal structures in adult crustaceans. The oceans are predicted to experience dramatic shifts in trophic structure^58^. Coral reefs, in particular, are likely to suffer structural disturbances leading to irreversible habitat loss and a decrease in both vertebrate and invertebrate diversity^58^. Smashing mantis shrimp are generalist predators that consume a diversity of prey^13^. With their predatory appendages intact and their broad diets, smashing mantis shrimp will likely be resistant to predicted near-term ocean conditions and continue to be high performing predators in the intertidal habitats of the future.

## Materials and Methods

### Animal acquisition and care

One hundred adult *Neogonodactylus bredini* [Crustacea: Stomatopoda: Gonodactylidae^59^] were captured by-hand between January 21–25, 2014 in the coral rubble and sea grass beds of the intertidal back reef at Galeta Marine Reserve, Smithsonian Tropical Research Institute (STRI), Colon, Panama (Autoridad Nacional del Medio Ambiente, Permit No. SEX/A-133-08). Animals were housed at the Naos Marine Laboratory, STRI, Panama City, Panama until February 25, 2014, when they were shipped to Scripps Institution of Oceanography (SIO), University of California, San Diego (UCSD). Animals were placed in individual 950 mL plastic cups (32 oz HDPE White Canister, 89 mm neck, United States Plastic Corp., Lima, Ohio, USA) that were connected to a flow through experimental aquarium system (see “Experimental design and maintenance” below for details). Individuals were acclimated to laboratory conditions for 37 days prior to the start of the experiment.

Individuals were fed 3–5 Pacific mole crabs (*Emerita analoga*) three times per week throughout captivity and the experiment. Any mole crabs that were not consumed after 5–12 hours were removed to prevent contamination from decaying crab material. Given that individuals usually had remaining mole crabs in their cups, we considered the feeding schedule to be *ad-libitum*.

### Experimental design and maintenance

The experimental ocean acidification system consisted of three 60.5 L reservoir tanks that served as one of the following three seawater treatments: ambient pH and ambient temperature (pH 7.88 ± 0.003, pCO_2_ = 636 ± 80 μatm, 27.4 ± 0.1 °C), reduced pH and ambient temperature (pH 7.57 ± 0.003, pCO_2_ = 1,335 ± 185 μatm, 27.2 ± 0.1 °C), and combined reduced pH and increased temperature (pH 7.59 ± 0.005, pCO_2_ = 1,301 ± 130 μatm, 29.7 ± 0.1 °C) (pH values are reported on the total seawater scale, pH_T_; see Supplementary Table S1 for additional water parameters). The reservoir tanks received filtered seawater pumped from the SIO Pier at ambient pH (7.9–8.2; data from Kram, S. L., Takeshita, Y., Dickson, A., Martz, T., & Smith, J. E. Scripps Ocean Acidification Real-time (SOAR) Dataset, Scripps Institution of Oceanography) and salinity (230–235 PSU) and warmed to 25 °C via heating plates. The temperature in all reservoir tanks was elevated to 27 °C with 300-W heaters to reflect temperatures at the collection site (data from the Smithsonian Tropical Research Institute’s Physical Monitoring Program). To further increase the temperature in the reduced pH/increased temperature treatment, an additional 800-W heater was placed in the reservoir tank, which was also wrapped externally in insulation material. One 1,609 L/h aquarium pump was placed in each reservoir tank five weeks after the start of the experiment to reduce variability in pH and temperature and maintain appropriate air saturation. Both pH and temperature in the reservoir tanks were constantly sampled and controlled (data logged every 20 min) using an Apex Lite aquarium controller and data logger (Neptune Systems, Morgan Hill, CA, USA) with on/off control of the aquarium heaters and the CO_2_ injection system (a tank of compressed CO_2_ gas controlled with a regulator and a solenoid valve). Each reservoir tank fed flow-through seawater to 24 cups containing individual animals (72 animals total). The flow rate to all experimental cups was 38 L/hr and uniformly maintained using sprinkler heads downstream of the header tanks (Xeri-Bird 8 multi-outlet emission device, Rain Bird, Azusa, CA, USA).

Prior to manipulating seawater conditions, 72 healthy adult *N. bredini* were selected. Each of these individuals was semi-randomly assigned to one of the three seawater treatments. Caution was given to maintain the same average body size and distribution of males and females across treatments (males: *N* = 22, females: *N* = 50). Water parameters were then changed incrementally over the course of a week to reduce stress to the animals. Once the desired water parameters were reached, the experiment was run for six months (24 weeks), with eight animals per treatment (24 animals total) being randomly subsampled at three months (12 weeks) (herein referred to as three-month and six-month exposure periods). The sample sizes were based on Taylor *et al*.^27^.

### Water chemistry

In addition to continuous monitoring with the CO_2_ controller system, both pH and temperature were recorded daily, from each reservoir tank and experimental cup using a portable probe (HQd, pH201e, Hach, Loveland, CO, USA), but only data from the experimental cups are reported (155 measurements per cup; measurements missing for 13 of the 168 days of the experiment; Supplementary Table S1). The portable probe and the probes used to control the reservoir tanks were recalibrated with standard buffer solutions every month and 1–2 weeks, respectively, to prevent probe drift.

Water samples were taken from each reservoir tank and from a subsample of experimental cups (one consistent experimental cup and one randomly selected cup from each treatment) once every two weeks and submitted to the Dickson Laboratory at SIO for analysis of total alkalinity, pH, and density-based salinity (Supplementary Table S1). pCO_2_, HCO_3_-, ΩCa, and ΩAr were calculated using CO_2_Sys v.2.1. pH values recorded on the NBS scale with the portable probe were converted to the total seawater scale using the known pH values from the water samples and CO_2_Sys. Thus, all pH values are reported on the total seawater scale. Water parameters remained consistent for the duration of the experiment (Supplementary Table S1).

To determine how experimental treatments compared to the range of values that mantis shrimp experience in the field, we returned to the collecting sites between March 7–25, 2015 (dry season) to document pH, temperature, salinity, and alkalinity (see Supplementary Information for details).

### Growth, molting, and mortality

Before the start of the experiment and on the day of sacrifice, total body length (measured from the tip of the rostrum to the tip of the telson), carapace length, and merus length of the raptorial appendage were measured (see Fig. 1 for anatomy). At the start of the experiment, animals ranged in total body length from 33.47 to 52.40 mm and in carapace length from 7.55 to11.95 mm. Mass ranged from 0.69 to 2.60 g. Animals were checked daily for deaths and molts. Molts were recorded and the exuviae were promptly collected throughout the experiment.

### Oxidative stress

To determine induced oxidative stress in individuals, we examined protein damage due to oxidative stress and the activity levels of two enzymes that are produced in response to high levels of oxidative stress: SOD and CAT. Reactive oxygen species (ROS) are produced as natural by-products of metabolism, but they can damage cells when produced in high quantities in response to increased metabolic activity, which is usually stress induced. For example, ROS can lead to the addition of carbonyl groups to proteins that harm protein function. SOD and CAT catalyze the breakdown of common ROS to help to reduce damage to proteins. These enzymes are particularly well-studied because they are important for coping with physiological damage due to oxidative stress across eukaryotes (reviewed in ref. 60). After six months, we tested for relative differences in the concentrations of proteins with carbonyl bond additions, SOD, and CAT as proxies for stress in muscle tissue from a subset of the experimental animals (3 individuals per treatment).

Degradation of these tissues may have occurred during storage in −20 °C for 5 months after the animals were sacrificed at the end of the six-month exposure. We assume, however, that the rate of degradation was the same across all experimental treatments, because all tissues were treated in the same manner. To ensure that tissue proteins were still intact, we conducted a protein gel electrophoresis (SDS-PAGE) with Coomassie Simply Blue staining on one tissue homogenate per individual, which revealed no detectable protein damage (Supplementary Fig. S2). This analysis confirmed that the tissues showed limited degradation while in storage.

We compared the enzyme activity and protein carbonyl concentration of the tissues from these experimental animals to those from six live individuals, three of which were maintained at pH 7.3 and the other three of which were maintained at ambient pH of 7.9 for one week (hereafter referred to as 0.25 month exposure). This comparison allowed us to determine whether there were differences in oxidative stress when individuals were placed at a very low pH and between short and long exposure periods. These six animals were then euthanized by flash freezing them in liquid nitrogen.

Muscle tissues from all samples were dissected from the abdominal segments, weighed, and then immediately placed in −80 °C for 24 hr to deeply freeze them before homogenization. Frozen tissues were homogenized by first grinding them into a fine powder with a mortar and pestle over dry ice. Samples were then transferred to a glass homogenizer with 1.2 mL of homogenizing buffer (phosphate buffer with 250 mM sucrose, 1 mM EDTA, 30 mM Tris, pH 7.52), protease inhibitor (P1860 Sigma, Aigma-Aldrich, St. Louis, MO, USA), and PhosSTOP (Roche Diagnostics Corporation, Indianapolis, IN, USA), manually homogenized, divided into twelve 100 μL aliquots, and frozen at −80 °C.

Bradford assays to determine protein concentration were performed on each sample homogenate used in the analyses of enzyme activity and protein carbonyl damage (see Supplementary Information for details).

Protein carbonyl abundance was measured using an OxiSelect Protein Carbonyl ELISA Kit (Cat. STA-310, Cell Biolabs Inc., San Diego, CA, USA) according to the manufacturer’s protocol. The plate was read immediately at 450 nm using the Spectramax i3. These values were processed with a linear regression generated from the standard curve (r^2^ = 0.98).

SOD and CAT enzyme abundances were measured with a SOD Colorimetric Activity Kit (Cat. K028-H1, Arbor Assays, Ann Arbor, MI, USA) and a CAT Activity Kit (Cat. K033-H1, Arbor Assays, Ann Arbor, MI, USA), respectively, following the manufacturer’s protocols. Using a Spectramax i3 (Molecular Devices, Sunnyvale, CA, USA), absorbance of the samples was read at 450 nm and 560 nm for SOD and CAT, respectively. Absorbance values of both SOD and CAT were normalized based on the protein concentrations measured from the Bradford assays. These values were then processed using a 4-parameter logistics curve generated from the standard curve.

### Exoskeleton sample preparation

At three months and six months of the experiment, eight and 13 individuals, respectively, were randomly selected from each treatment to be measured and weighed following the procedures described above and then euthanized by brief placement in a −20 °C freezer until visible movement ceased. Individuals were sampled at least 18 days following ecdysis (range: 18–171 days) because the exoskeleton is fully hardened at this time in *N. bredini*^46^. Samples of cuticle were dissected from the lateral side of the merus and the lateral flap of the carapace (Fig. 1). One appendage and one side of the carapace were randomly selected for morphological analysis using SEM while the opposite appendage and side of carapace was used for materials testing. Cuticle samples were cleaned with a small paintbrush to remove the underlying hypodermis and then immediately placed in seawater.

One additional individual was selected from each treatment at the end of the six-month exposure for Micro-Computed Tomography scans (Micro-CT scans). These individuals were promptly euthanized and transferred to the Micro-CT scanner.

### Scanning electron microscopy

The morphology and mineral content of cuticle samples were analyzed with SEM and EDX at the Nano3 Facility at UCSD. Cuticle samples were freeze-fractured with liquid nitrogen, placed in a critical point drier (AutoSamdri 815 Series A, Tousimis, Rockville, MD, USA), secured to a double 90° SEM mount revealing the cross-section, and sputter coated with iridium.

Cross-sections of cuticle samples were examined with an ultra-high resolution SEM equipped with EDX (XL30 SFEG with Sirion column, Field Emission Incorporated, Hillsboro, OR, USA). SEM imaging was done at 10 kV acceleration voltage and four images from each segment were taken from separate locations on each merus and carapace cuticle sample. Total cuticle thickness (epicuticle, exocuticle, and endocuticle) was measured from each image. The membranous layer was excluded from this measurement because it was not visible.

Mineral composition and distribution were examined in both the merus and carapace cuticle samples using EDX. While Ca content is typically estimated from the dry weight of the Ca in the calcified structure (methods based on^31^,^61^), we used EDX to quantify the relative atomic and weight percent of elements across a focal 2-dimensional surface^23^,^27^. EDX has been shown to successfully quantify mineral content in crustacean exoskeleton^23^, including the mantis shrimp dactyl club^24^ and caridean shrimp carapace^27^. Each cuticle image was magnified so that it filled the computer screen. The spectra and element maps were all collected at a 20 kV acceleration voltage and a minimum of 5,000 counts per second. A semi-quantitative analysis of all elements in the cuticle cross-section was conducted using EDX spectra from up to two cuticle locations on each sample. Calcium, magnesium, carbon, and oxygen were the elements typically found in larger amounts. Aluminum, chlorine, phosphorus, and sodium were also sometimes present in smaller amounts (Supplementary Fig. S3). The amount of calcium and magnesium in each sample was calculated as the weight percent (wt %) and atomic percent (at %) relative to all detected elements, excluding the iridium coating. In addition, EDX element maps were taken from one location on each sample (from only one individual per treatment) and used to visualize the distribution and density of calcium and magnesium in the cuticle cross-section (Supplementary Fig. S1).

### Micro-CT scans

To further analyze mineral densities of targeted sections of the raptorial appendage and carapace, Micro-CT scans were performed at the Cartilage Tissue Engineering Lab at UCSD. The three frozen mantis shrimp were thawed and then imaged with a Micro-CT scanner (Skyscan 1076, Kontich, Belgium) in a humidified environment to prevent sample dehydration during scanning. Imaging was performed at 9 μm isotropic voxel size by applying an electrical potential of 50 kVp and current of 200 μA, and using a 0.5 mm aluminum filter. A beam hardening correction algorithm was applied during image reconstruction. Mineral density was determined by calibration of images against 2 mm diameter hydroxyapatite (HA) rods (0.25 and 0.75 gHA/cm^3^). To visualize and determine histomorphometric parameters, the software programs, Dataviewer, CTAn, and CTVox (all Skyscan, Kontich, Belgium) were used.

Mineral densities of merus and carapace sections from the right side of the appendage were quantified using a slice-by-slice 2D analysis and integrating all slices over the region of interest. Specifically, 10% of the total volume of the right flap of the carapace, and 20% of the total volume of the lateral side of the merus were analyzed (Supplementary Fig. S4). To determine if the appendage’s striking surface was affected by changes of the experimental treatment, the mineral densities of the rounded portion of the dactyl club (Fig. 1) were analyzed using a 3D analysis based on dactyl club volume (Supplementary Fig. S4).

To segment out volumes of interest for quantification, a global threshold was used, and an erosion of one pixel was performed to eliminate partial volume effects. For the merus and carapace, the following parameters were determined over the full volume: cross-sectional total area (T.A.), cross- sectional object area (Obj.A.), object area fraction (Obj.A./T.A.), object thickness (Obj.Th), and tissue (or true) mineral density (T.M.D.). For the dactyl club, the following parameters were determined: total volume (T.V.), object volume (Obj.V.), object volume fraction (Obj.V./T.V.), object surface (Obj.S.), and tissue mineral density (T.M.D.).

### Mechanical properties

The complementary merus and carapace cuticle samples removed from the experimental animals, as described above, were tested for hardness and stiffness (i.e. elastic modulus) immediately after animals were sacrificed using a nanoindentation materials testing machine (Nano Hardness Tester, Nanovea, Irvine, CA, USA) equipped with a Birkovich diamond indenter tip. Flat sections of the merus and carapace were dissected from the posterior ends of the cuticle samples and then mounted with super glue onto a steel block with the epicuticle exposed. All indentations were performed by penetrating through the epicuticle layer with a load of 80 mN, loading and unloading rates of 160 mN/min, and 30 sec of creep. A total of 3–5 indents were made on each cuticle sample.

### Statistics

All data were tested for normality using the Shapiro-Wilk test and for homogeneity of variances using Bartlett’s test. Statistical analyses were conducted on the log-transformed concentrations of SOD, CAT, and proteins with carbonyl bonds. Mean SOD values in individuals from the ambient and reduced pH treatments from the 0.25-month exposure were compared to SOD values in individuals from the ambient, reduced pH, and reduced pH/increased temperature treatments from the six-month exposure using ANOVA. The log-transformed CAT and protein carbonyl data were not normally distributed and a Kruskal-Wallis non-parametric test was therefore used to evaluate differences between treatments.

The means of percent growth (mass and carapace length), cuticle thickness (corrected for body size), weight percent calcium, weight percent magnesium, the ratio of calcium to magnesium, cuticle hardness, and cuticle stiffness were compared across animal size and between treatments. Percent growth variables were log-transformed prior to analyses to be consistent with the other data sets (the arc-sign transformed data produced the same results). Growth, morphological, and mechanical properties data were normally distributed and homogeneous. One-way ANOVA’s were used to compare growth between treatments. Between treatments, appendage segments, and exposure periods (three vs six months) comparisons of morphological and mechanical properties data were conducted using three-way ANOVA’s. Two-way ANOVA’s were used to evaluate the difference between treatments and exposure times within either the merus or the carapace. Least-squares linear regression analyses were used to evaluate scaling patterns in the morphological and mechanical property variables across animal size, and an ANCOVA was used to test for differences between treatments with carapace length as the co-variate. An ANCOVA analysis was also used to examine differences between treatments and the effect of time since the last molt on all response variables using a Bonferroni adjusted α = 0.004.

All descriptive statistics (*N* values, mean ± standard deviation, and mean difference [95% confidence interval]) and statistical tests were performed in R version 3.1.2^62^.

## Additional Information

**How to cite this article:** deVries, M. S. *et al*. Stress physiology and weapon integrity of intertidal mantis shrimp under future ocean conditions. *Sci. Rep.* **6**, 38637; doi: 10.1038/srep38637 (2016).

**Publisher's note:** Springer Nature remains neutral with regard to jurisdictional claims in published maps and institutional affiliations.

## Supplementary Material

Supplementary Information

## Figures and Tables

**Table 1:** Concentrations of enzymes and proteins with carbonyl bonds as indicators of oxidative stress.

Treatment	Exposure period	SOD (U/mL × mg)		CAT (U/mL × mg)		Protein carbonyl (nmol/mg)	
		Mean	Range	Mean	Range	Mean	Range
Ambient (pH 7.9)	0.25 month	0.19 ± 0.02	0.17–0.21	0.67 ± 0.07	0.60–0.70	0.63 ± 0.03	0.60–0.66
Low (pH 7.3)	0.25 month	0.21 ± 0.01	0.21–0.22	0.68 ± 0.03	0.64–0.70	0.41 ± 0.24	0.26–0.68
Ambient (pH 7.9)	6 months	0.19 ± 0.02	0.17–0.21	0.58 ± 0.18	0.37–0.69	0.48 ± 0.14	0.37–0.64
Reduced (pH 7.6)	6 months	0.18 ± 0.02	0.15–0.20	0.55 ± 0.14	0.41–0.68	0.34 ± 0.07	0.26–0.40
Reduced (pH 7.6), increased temp	6 months	0.20 ± 0.02	0.18–0.21	0.66 ± 0.05	0.58–0.69	0.36 ± 0.12	0.26–0.54

Values are mean ± standard deviation concentration and concentration range (min-max) of superoxide dismutase (SOD), catalase (CAT), and protein carbonyl bonds (protein carbonyl) from individuals from a 0.25-month exposure and a six-month exposure. *N* = 3 for each concentration presented, U = activity level.

**Table 2:** Exoskeleton thickness, mineral composition, and mechanical properties.

Segment	Exposure period	Treatment	Thickness*	Ca (wt. %)	Mg (wt. %)	Ca:Mg	Hardness (GPa)	Stiffness (GPa)
merus	3 months	ambient	8.62 ± 1.34 (*N* = 6)	35.38 ± 6.52 (*N* = 6)	2.86 ± 0.25 (*N* = 6)	12.47 ± 2.80 (*N* = 6)	0.48 ± 0.03 (N = 7)	6.41 ± 0.48 (N = 7)
		reduced pH	9.91 ± 1.6 (*N* = 8)	41.13 ± 10.47 (*N* = 7)	**2.99 ± 0.71** (*N* = 7)	14.50 ± 5.14 (*N* = 7)	0.49 ± 0.02 (N = 8)	4.98 ± 0.73 (N = 8)
		reduced pH/increased temp	9.21 ± 0.72 (*N* = 7)	34.74 ± 1.96 (*N* = 6)	2.83 ± 0.33 (*N* = 6)	12.43 ± 1.52 (*N* = 6)	0.47 ± 0.07 (N = 8)	4.14 ± 0.92 (N = 8)
	6 months	ambient	9.41 ± 1.36 (*N* = 12)	*46.46 *±* 6.19 (N* = 11)	2.62 ± 0.58 (N = 11)	*19.03 *±* 7.52* (N = 11)	0.51 ± 0.07 (N = 12)	5.18 ± 1.07 (N = 12)
		reduced pH	9.38 ± 2.22 (*N* = 11)	*44.28 *±* 5.21 (N* = 11)	**3.37 ± 0.81** (N = 11)	*13.90 *±* 3.68* (N = 11)	0.51 ± 0.05 (N = 10)	4.52 ± 0.19 (N = 10)
		reduced pH/increased temp	9.20 ± 1.70 (*N* = 12)	*46.84 *±* 7.18 (N* = 12)	2.57 ± 0.51 (N = 12)	*19.16 *±* 5.70* (N = 12)	0.57 ± 0.07 (N = 11)	6.70 ± 1.38 (N = 11)
carapace	3 months	ambient	9.84 ± 1.66 (*N* = 8)	37.67 ± 5.73 (*N* = 6)	2.36 ± 0.42 (N = 6)	16.61 ± 5.51 (N = 6)	0.38 ± 0.02 (N = 8)	6.12 ± 0.24 (N = 8)
		reduced pH	11.84 ± 1.57 (*N* = 6)	35.54 ± 3.93 (*N* = 5)	2.95 ± 0.71 (N = 5)	12.93 ± 4.69 (N = 5)	0.39 ± 0.05 (N = 7)	2.99 ± 0.79 (N = 7)
		reduced pH/increased temp	10.54 ± 1.54 (*N* = 7)	32.95 ± 6.50 (*N* = 7)	2.52 ± 0.51 (N = 7)	13.75 ± 4.48 (N = 7)	0.43 ± 0.05 (N = 8)	5.62 ± 0.67 (N = 8)
	6 months	ambient	9.94 ± 1.11 (*N* = 12)	*41.33 *±* 8.32 (N* = 11)	3.01 ± 0.61 (N = 11)	14.37 ± 4.61 (N = 11)	*0.48 *±* 0.09* (N = 10)	*6.84 *±* 0.80* (N = 10)
		reduced pH	10.57 ± 2.38 (*N* = 11)	*39.16 *±* 7.13 (N* = 11)	3.18 ± 0.60 (N = 11)	12.64 ± 2.94 (N = 11)	*0.55 *±* 0.09* (N = 11)	*10.18 *±* 1.32* (N = 11)
		reduced pH/increased temp	10.95 ± 1.94 (*N* = 11)	*40.41 *±* 6.69 (N* = 12)	3.04 ± 0.70 (N = 12)	13.80 ± 3.56 (N = 12)	*0.51 *±* 0.05* (N = 11)	*8.62 *±* 0.83* (N = 11)

Values are mean ± standard deviation. *N* = number of individuals. Bold values indicate significant difference between treatments (see text for statistics). Values in *italics* indicate significant difference between exposure periods within treatments (α = 0.05). *Thickness is unitless, as it was corrected for body size by dividing values by carapace length.

**Table 3:** Mineral density as calculated from the Micro-CT scans.

Segment	Treatment	T.A. (mm^2^) or T.V. (mm^3^)*	Obj.A. (mm^2^) or Obj.V. (mm^3^)*	Obj.A./T.A. or Obj.V./T.V.* (%)	Obj.Th. (mm) or Obj.S. (mm^2^)*	T. M. D. (gHA/cm^3^)
merus	ambient	11.734	0.368	3.13	0.063	0.631
	reduced pH	11.734	0.489	4.17	0.076	0.694
	reduced pH/increased temp	11.734	0.387	3.30	0.062	0.663
carapace	ambient	5.056	0.191	3.78	0.061	0.562
	reduced pH	5.056	0.186	3.67	0.063	0.634
	reduced pH/increased temp	5.056	0.155	3.06	0.058	0.613
dactyl*						
low density	ambient	2.997	2.089	69.70	14.359	1.294
	reduced pH	3.063	2.019	65.91	14.132	1.344
	reduced pH/increased temp	2.844	1.490	52.38	17.929	1.455
high density	ambient	2.997	0.747	24.91	12.488	1.877
	reduced pH	3.063	0.879	28.70	13.978	1.879
	reduced pH/increased temp	2.844	1.011	35.54	25.348	1.853

The carapace and merus were quantified via a 2D analysis by integrating 2D slices in the region of interest as defined by the total area (T.A.), while the dactyl was 3D analyzed in the total volume (T.V.) of interest. Area metrics presented refer to the merus and carapace. Volume metrics refer to the dactyl as indicated by the*. Obj.A or Obj.V. is the total object area or volume obtained inside the T.A. or T.V., respectively. Obj.A.**/**T.A. is the object area divided by the total area, and Obj.V.**/**T.V. is the object volume divided by the total volume; both metrics have the same units. Obj.Th. is the thickness of the object and Obj.S. is the surface area of the object’s volume. T.M.D. is the true mineral density calculated by correlating attenuation coefficients from images of sample and hydroxyapatite (HA) rods of known densities. The units for T.M.D are the same for the merus, carapace, and dactyl.
