# Supplementary material for: Stress physiology and weapon integrity of intertidal mantis shrimp under future ocean conditions
Source: Sci Rep. 2016 Dec 15;6:38637. doi: 10.1038/srep38637 (PMC5156921; doi:10.1038/srep38637)
Supplement: Supplementary Information [file srep38637-s1.pdf]

SUPPLEMENTARY INFORMATION

Stress physiology and weapon integrity of intertidal mantis shrimp under future ocean conditions

Maya S. deVries[1*], Summer J. Webb[1], Jenny Tu[1], Esther Cory[2], Victoria Morgan[1], Robert L. Sah[2], Dimitri D. Deheyn[1], & Jennifer R. A. Taylor[1]

[1] Scripps Institution of Oceanography, Marine Biology Research Division, University of California at San Diego, La Jolla, CA 92093 U.S.A.

[2] Department of Bioengineering, University of California at San Diego, La Jolla, CA 92093 U.S.A.

* Corresponding author: mdevries@ucsd.edu

SUPPLEMENTARY METHODS

Field pH and temperature data collection

To record pH and temperature in the field, we used a portable pH probe (NBS values subsequently converted to the total seawater scale) in the sea grass beds from which *N. bredini* individuals were collected at two main collecting sites, Isla Mina and Galeta Marine Laboratory from March 7-11 and March 20-24 (10 days total). We focused on the sea grass specifically because our goal was to capture the range of values experienced by *N. bredini*, and sea grass photosynthesis and respiration influences the pH environment. Thus, we recorded pH and temperature at high and low tides, and before sunrise and after midday to capture the variation in pH values that is created by sea grass respiration and photosynthesis. We also recorded these parameters at high tide, low tide, and after midday at Isla Mina, but we were unable to document pH and temperature before sunrise because this site was too remote to access at night. On March 24, 2015, two water samples were collected at three time points: before sunrise, at low tide, and after midday (6 samples total) at Galeta Marine Laboratory, and three water samples were collected after midday at Isla Mina. All samples were brought to the Dickson Laboratory at Scripps Institution of Oceanography for analysis of pH, total alkalinity, and density based salinity, as described in the Materials and Methods section of the main manuscript.

Oxidative stress

<u>Bradford assays</u>: To perform the Bradford assays, 200 µL of 1X Bradford dye (Bio-Rad Laboratories, Hercules, CA) and 10 µL of either the sample or bovine serum albumen (BSA) standard were added to a well plate. Samples were measured using an iMark Microplate Absorbance Reader (Bio-Rad Laboratories, Hercules, CA) at 595 nm. Standard curves were generated using BSA at 0.9, 0.7, 0.56, 0.28, 0.14, and 0 µg/µL protein concentrations (all R^2-values were > 0.95) to calculate sample concentrations based on the best fit line of the standard curves.

<u>Protein carbonyl analysis</u>: The protein concentrations of the samples were first determined with the Bradford Assay. The samples were then diluted to 10 µL/mL based on protein concentrations. Samples were compared to BSA standards. Standards and samples were plated into a protein binding plate in triplicate and incubated overnight. 100 µL of DNPH solution, 200 µL of blocking solution, 100 µL of anti-DNP antibody, and 100 µL of HRP secondary antibody was added to and removed from the binding plate. Three to five washes with 200 µL of either 1X PBS solution or wash solution were interspersed between each step. 100 µL of substrate solution was added to the plate and 100 µL of stop solution was added after 15 min.

<u>Superoxide dismutase (SOD) and catalase (CAT) analyses</u>: To analyze SOD enzyme abundance, 50 µL of the substrate preparation was combined with 10 µL of the *N. bredini* tissue homogenate, and then 25 µL of xanthine oxidase preparation. Samples were prepared in triplicate and compared to BSA standards. Prior to reading, the plate was centrifuged at 3,221 g for 3 min and incubated at room temperature for 20 min. CAT abundance was measured with a CAT Activity Kit following similar methods (25 µL of sample, 25 µL of H_2O_2, and 25 µL of detection reagent, and 25 µL of substrate). Samples were prepared in triplicate and compared to bovine CAT as standards. Prior to reading, the plate was centrifuged at 3,000 g for 1 min and incubated at room temperature for 15 min.

SUPPLEMENTARY TABLE

Supplementary Table S1. Water chemistry of samples from the laboratory and field. Values are mean ± standard deviation of chemical properties of the seawater in the experimental cups over the course of the six-month exposure period and of seawater samples from the two collecting sites, Galeta Marine Lab and Isla Mina, Panama. 155 measurements were taken per experimental cup and the mean pH and temperature values for each cup was then used to calculate the overall means for each treatment. Measurements at Galeta Marine Lab were taken throughout the day and night but only times with the extremes of the measurement range are presented (high tide before sunrise and low tide in the afternoon). For Isla Mina, measurements at low tide in the afternoon are presented. pH, salinity, and total alkalinity (TA) were measured by the Dickson Laboratory and all other parameters were calculated using CO2sys. pH_T denotes the total seawater scale. * Indicates significant differences between experimental treatments ($\alpha = 0.05$). Note that while the pH values of the cups were significantly different between treatments, the mean difference [95% C.I.] between the reduced pH and reduced pH/increased temperature treatments was only 0.02 [0.02, 0.02] and the total alkalinity, pCO_2, HCO_3^-, and ΩCa were not significantly different. The temperature of the experimental cups was significantly different between treatments, but the mean difference between the ambient and reduced pH treatments was only 0.24°C, which we do not think is biologically relevant.

Experiment / Collecting sites	Treatment	Temperature (°C)	pH_T	Salinity (%)	TA (μmol/kgSW)	HCO_3^- (μmol/kgSW)	pCO_2 (μatm)	ΩCa	ΩAr
Experiment (*N*=72)	ambient	27.41 * ± 0.05	7.88 * ± 0.003	33.61 ± 0.04	2,235.98 ± 3.87	1,849.96 ± 34.92	635.51 ± 79.94	3.85 ± 0.33	2.55 ± 0.23
	reduced pH	27.17 * ± 0.08	7.57 * ± 0.003	33.61 ± 0.05	2,236.53 ± 4.57	2,019.41 ± 22.78	1,334.84 ± 184.70	2.17 ± 0.23	1.44 ± 0.15
	reduced pH/ increased temp	29.67 * ± 0.12	7.59 * ± 0.005	33.61 ± 0.04	2,236.19 ± 3.13	1,998.98 ± 17.76	1,300.88 ± 130.23	2.39 ± 0.18	1.60 ± 0.12
Galeta Marine Lab (*N*=353)	high tide, pre-dawn	27.34 ± 0.13	7.94 ± 0.01	35.42 ± 0.18	2,267.01 ± 21.50	1,793.83 ± 27.02	495.05 ± 16.35	4.62 ± 0.06	3.07 ± 0.04
	low tide, afternoon	28.12 ± 0.44	8.32 ± 0.03	35.28 ± 0.20	2,189.65 ± 46.95	1,368.71 ± 246.42	216.01 ± 125.20	7.97 ± 1.90	5.36 ± 1.30
Isla Mina (*N*=117)	low tide, afternoon	28.67 ± 0.19	8.37 ± 0.03	35.10 ± 0.10	2,165.38 ± 35.10	1,199.95 ± 12.74	132.56 ± 4.23	9.36 ± 0.11	6.31 ± 0.08

SUPPLEMENTARY FIGURES

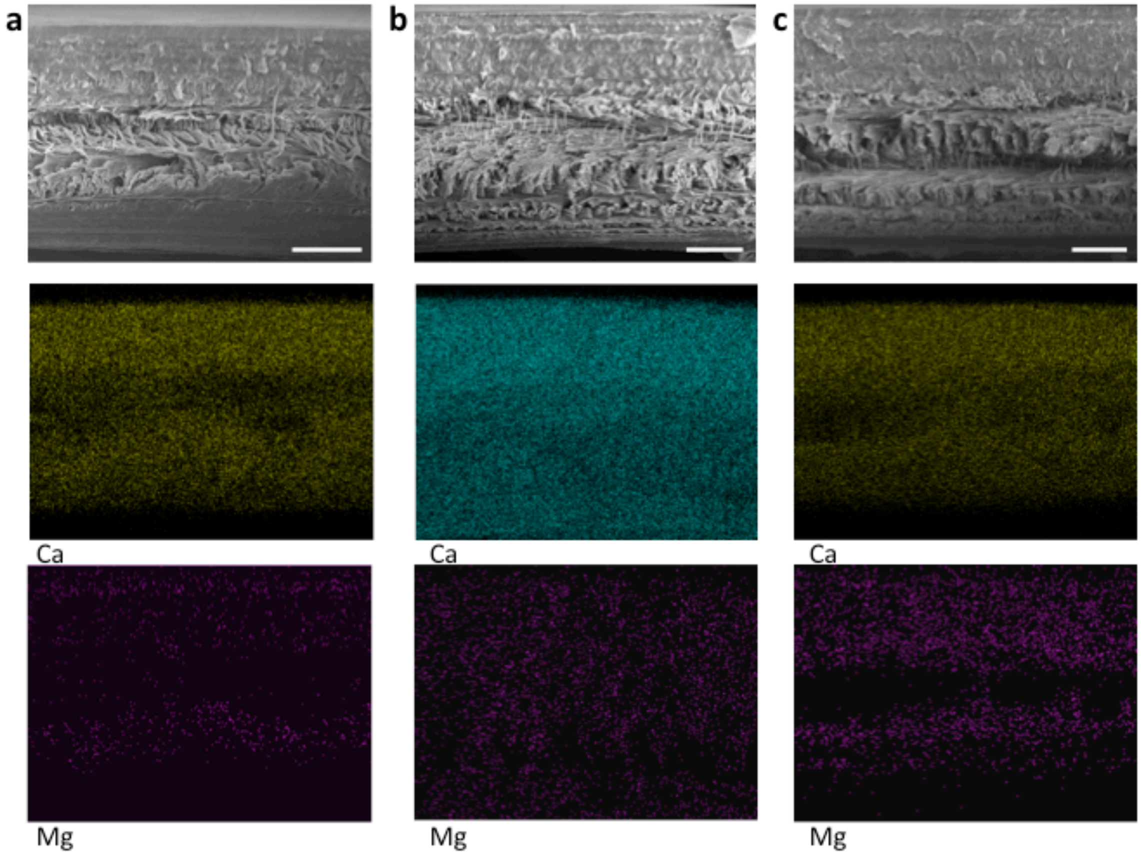

Supplementary Figure S1. Mineral maps of Ca and Mg distribution. Merus samples from (**a**) ambient, (**b**) reduced pH, and (**c**) reduced pH/increased temperature treatments are shown. The top row shows the SEM images of the mapped regions. The middle and bottom rows show the maps for Ca and Mg distributions, respectively, that were generated from the EDX analysis. Ca density appears to be uniform across the cuticle and across treatments. Mg density and distribution was higher in the reduced pH treatment compared to the other treatments. Maps for the carapace are not shown because the distribution of the elements in the samples was similar to the merus, although overall, the carapace was less dense than the merus. Scale bars = 20 μm.

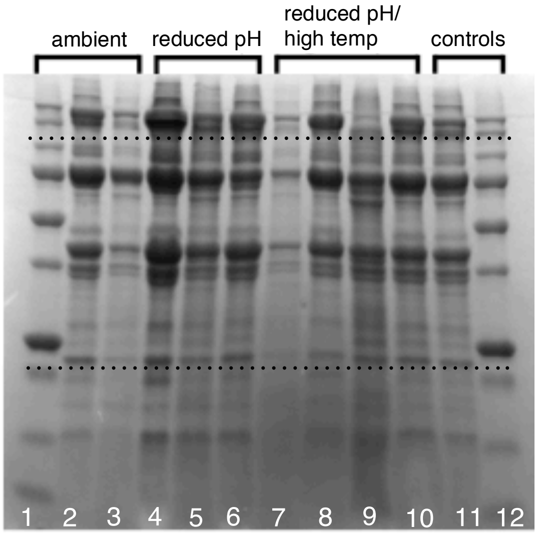

Supplementary Figure S2. SDS-Page gel electrophoresis of the experimental samples examined for oxidative stress. The gel image shows no protein degradation or "smear" indicating limited protein degradation. Each protein band (arranged in columns labeled 1-12) is a sample from a different individual that was in either the ambient, reduced pH, or reduced pH/increased temperature treatments. The columns labeled "controls" were animals that had been frozen and stored at -20°C and -80°C to examine whether the sample stored -20°C had noticeably more tissue degradation than the sample stored at -80°C. Dotted reference lines help compare migration of the bands in the gel and identify differences between bands that otherwise look similar (e.g. lanes 1 and 12). The well-defined bands in all samples suggest that the tissues were sufficiently intact to produce reliable results for protein assays (i.e. all tissues stored at -20°C experienced as much detectable tissue degradation as the control stored at -80°C). The intensity of the bands, therefore, does not reflect differences between storage at -20°C or-80°C. Rather, the differences in the levels of band migration in the gel and their respective intensities illustrate individual variation between samples.

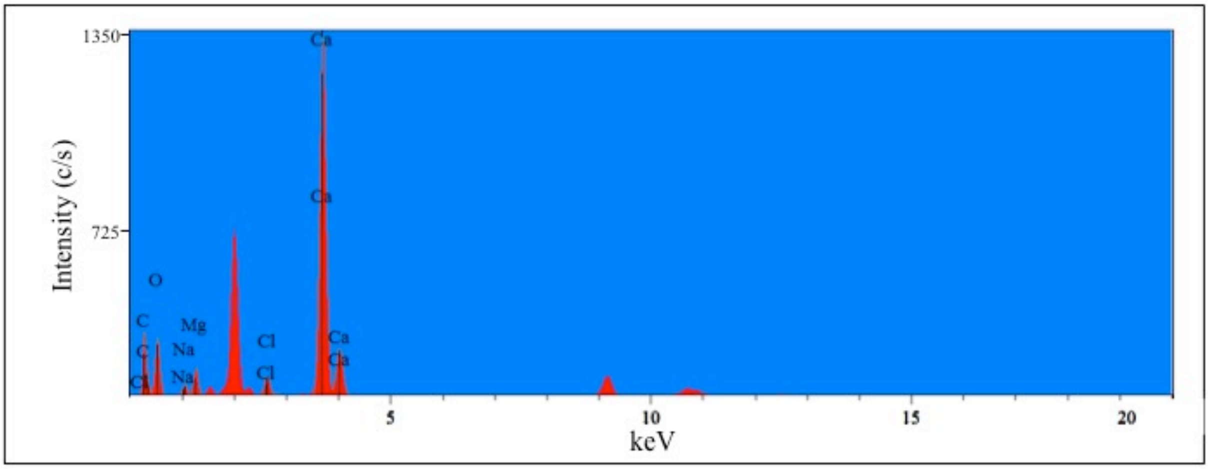

Supplementary Figure S3. Representative EDX spectrum from a cuticle cross-section. This spectrum is from the merus segment of an individual mantis shrimp in the ambient treatment. The spectrum was taken at 20 keV. Elemental peaks were consistently detected for C, O, Na, Mg, Cl, and Ca. Unlabeled peaks are Ir, which was used to coat the samples and were not included in the quantitative analysis. In this particular sample, % Ca and % Mg in relation to the other detected elements were 41.25 wt% and 2.69 wt%, respectively.

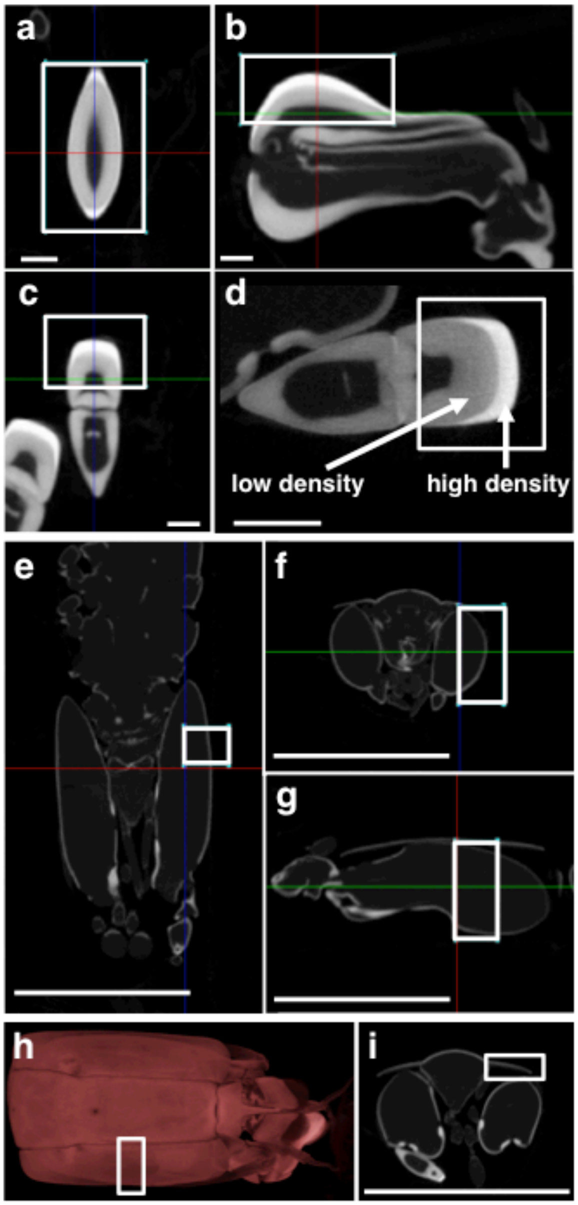

Supplementary Figure S4. Mineral density analysis from Micro-CT scans. Cross sections of the regions of the raptorial appendage and carapace analyzed for mineral density in Micro-CT scans. White boxes represent the volume quantified for the dactyl (**a**-**d**), merus (**e**-**g**), and carapace (**h**-**i**). Location of the 2D orthogonal cross-sections are indicated by the cross hairs and were used to determine the regions of interest. **Dactyl**: frontal (**a**), lateral (**b**), and ventral (**c**, **d**) views of the right dactyl are shown where the ventral view in (**d**) is a magnified view of that of (**c**) to highlight the high and low mineral density regions that were analyzed. **Merus**: ventral (**e**), frontal (**f**), and lateral (**g**) views of the right merus are shown. **Carapace**: 3D volume render of the Micro-CT scan of the dorsal view of the carapace (**h**) shows the region of interest analyzed on the right carapace flap, while the (**i**) shows the analyzed structure in cross-section. Scale bars = 1 mm for the dactyl and 1 cm for the merus and carapace.
